# Sacral Osteoneogenesis after Complete Sacrectomy in a Patient with Ewing Sarcoma

**DOI:** 10.1155/2017/7824687

**Published:** 2017-12-17

**Authors:** T. Hockertz, W. Eberl, M. Velickovic

**Affiliations:** ^1^Department of Orthopedic Surgery, Sports Traumatology and Trauma Surgery, Städtisches Klinikum Wolfenbüttel (Wolfenbüttel Municipal Hospital), Alter Weg 80, 38302 Wolfenbüttel, Germany; ^2^Hospital for Children's and Youth Medicine, Städtisches Klinikum Braunschweig (Braunschweig Municipal Hospital), Salzdahlumer Straße 90, 38126 Braunschweig, Germany

## Abstract

Ewing sarcomas are the second most common primary malignant bone tumors in childhood and adolescence which rapidly metastasize. Due to improvement of treatment options in recent years, the survival rate has significantly increased. Nevertheless, lethality is still high, and neurologic symptoms are frequent. To the best of our knowledge, this is the first reported case of a sacral osteoneogenesis after complete sacrectomy in a patient with Ewing sarcoma.

## 1. Introduction

Ewing sarcomas are the second most common primary malignant bone tumors in childhood and adolescence which rapidly metastasize. The femur and the pelvis are affected most frequently with approximately 20% of cases. In children under 15 years of age, the incidence is approximately 3 out of 1,000,000; in adolescents and young adults aged 15 to 25 years, the incidence is approximately 2.4 out of 1,000,000 in Germany. Due to improvement of treatment options in recent years, the survival rate has significantly increased. Key to success is an urgent and aggressive therapeutic algorithm [1, 2].

## 2. Presentation of Case

**Figure 1:**
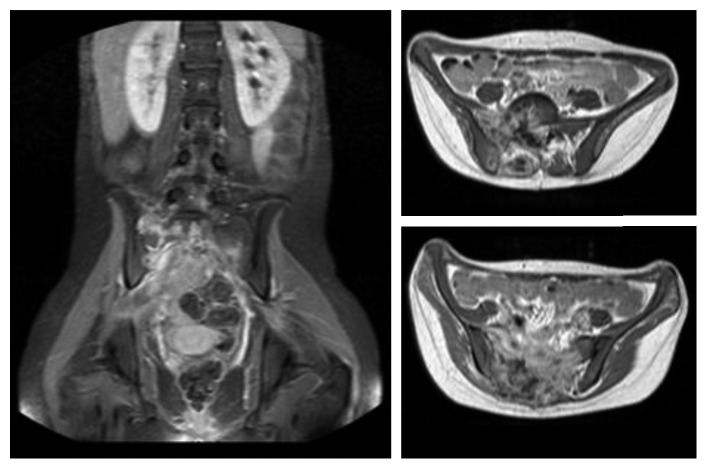
Selection of characteristic coronal and axial T1-weighted MRI slices of the Ewing Sarcoma.

**Figure 2:**
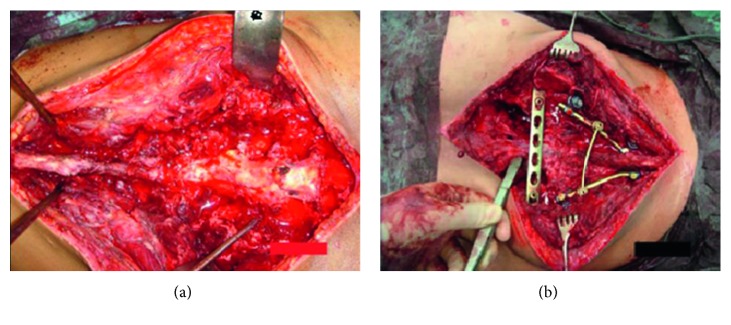
(a) Operative findings on the left; (b) on the right, there is the USS as well as the ilioiliac LCP visible.

**Figure 3:**
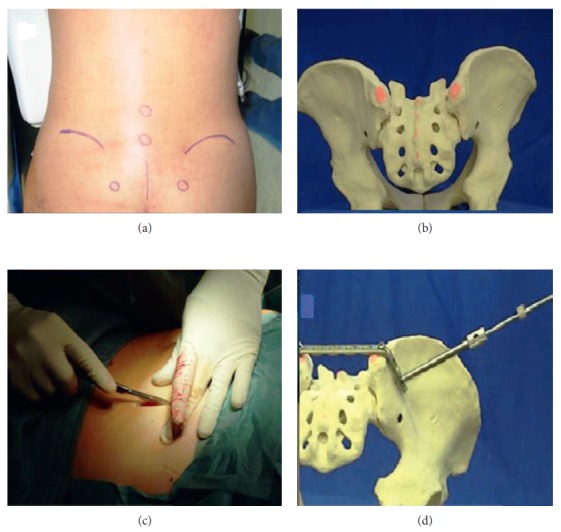
Operating sequence with (a) positioning of the patient, (b) marking of the landmarks, (c) surgical incision, and (d) setting of the angle-stable screws.

**Figure 4:**
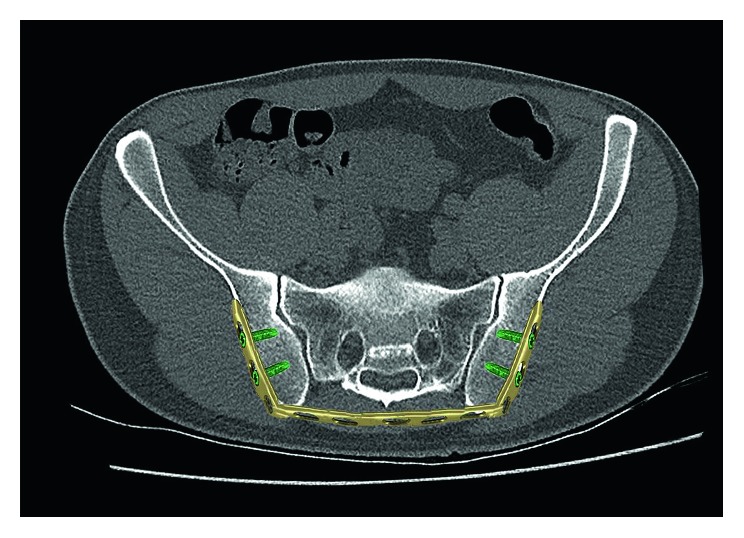
Schematic representation of the operation.

**Figure 5:**
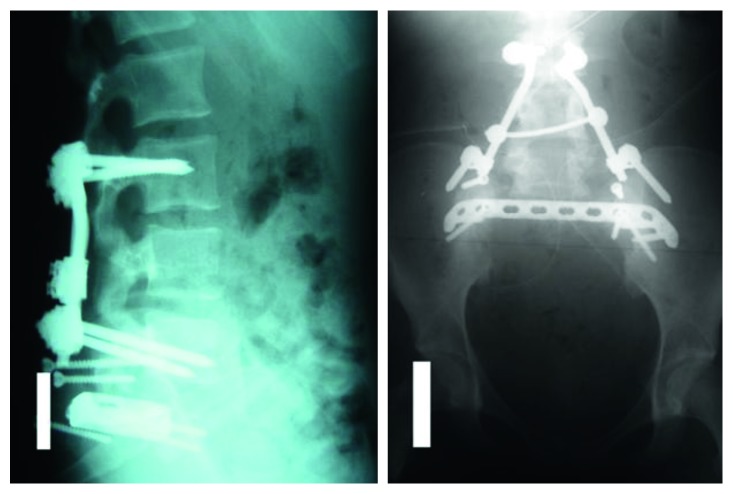
Postoperative X-rays of the lower spine in two axes (AP and lateral).

**Figure 6:**
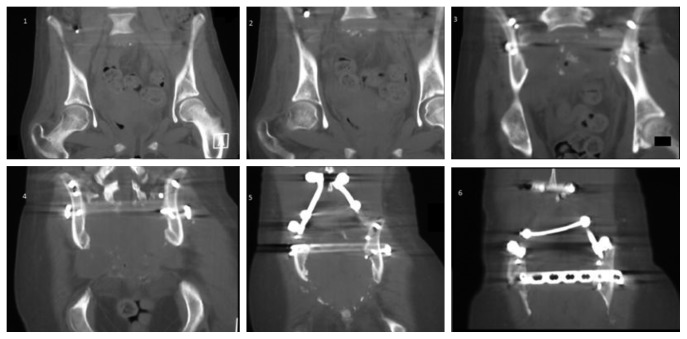
Postoperative CT (coronal slices) showing a complete removal of the sacrum.

The following case report is a long-term observation with a follow-up period of 12 years. An 18-year-old female presented in 2004 with a four-month history of progressive pain in the lumbosacral region radiating into both legs with saddle block anesthesia, foot flexor paresis, and episodes of bladder and stool incontinence. An MRI of the lumbar spine showed a shortened filum terminale up to the lower edge of the 3rd lumbar vertebral body which leads to conus fixation. Together with the clinical symptoms, a tethered cord syndrome was presumed. A surgery with microsurgical release of the conus to restore cord mobility was performed at a neurosurgical clinic. The patient did not benefit after the surgery, so the patient was retransferred to our hospital. In addition to the already existing symptoms, the patient complained about pain in the posterior pelvic ring; thus, an MRI of the pelvis was performed. Here, a partly intra- and extraosseous tumor of the pelvis with involvement of the entire right sacrum wing and extensive extraosseous growth in the small pelvis and into the dorsal pelvic soft tissue were diagnosed. In addition, there was an already diffuse intraspinal tumor spread throughout the sacral spinal canal extending cranially to the intervertebral disc level L5/S1 (Figure 1). The histopathological assessment of the sample biopsy revealed a malignant small- and bluish-cell tumor, corresponding to an Ewing sarcoma. Staging showed no evidence of metastasis of the tumor. The stage was documented with T3 N0 M0. An interdisciplinary therapy consisting of a combination of neoadjuvant chemotherapy, irradiation, and surgery was established. Within the scope of the imaging follow-up diagnostics, a good therapeutic response was shown with considerable reduction in the size of the tumor so that the surgery seemed promising. The tumor had an extension of 9 × 6.5 × 12 cm before the start of the therapy and 8 × 5.5 × 10.5 cm after 6 chemotherapy blocks (data in each case in width × depth × height). The surgery was performed in prone position, and the anatomical landmarks such as spina iliaca posterior superior and crista iliaca as well as the spinous processes of the lower lumbar spine were marked. In the first step, a posterior spondylodesis from the ileum to the third lumbar vertebra was performed with the USS (Universal Spine System; DePuy Synthes). The sacral spinal canal and neuroforaminae were decompressed. Because an en bloc resection of the tumor localization and spread seemed not possible, a step-by-step resection of the sacrum starting from the neuroforaminae was performed. Before complete sacral resection, a transiliac locking compression plate was additionally fixed in both iliac wings in order to ensure anatomical positioning of the pelvis (Figure 2). We have used a 11-hole LCP (Locking Compression Plate, DePuy Synthes) which was manually bent to the anatomic shape of the ilium. The horizontal orientation of the plate is controlled by X-rays and temporarily fixated with K-wires. Two angle-stable screws were subsequently screwed into the lateral plate holes after preliminary drilling and length measurement. The transiliac pelvic plate is routinely used in our hospital to treat traumatic and osteoporotic insufficiency fractures of the sacrum. The use in tumor orthopedics is a special indication [3]. After completion of the X-ray, rinsing of the surgical access and wound closure were performed (Figures 3 and 4). Postoperative X-ray and CT showed a complete removal of the sacrum (Figures 5 and 6). The operation lasted 8 hours. Due to the high blood loss, 26 erythrocyte concentrates as well as 14 fresh frozen plasmas and 4 thrombocyte concentrates were transfused. The tumor was completely removed macroscopically, and histopathological processing revealed total tumor necrosis without reference to vital tumor cells in all surgical preparations (Response Grad I according to Sulzer–Kuntschik). According to the R classification for the assessment of the resection margin of surgical preparations, this was an intralesional tumor resection so that irradiation was absolutely necessary. Immediately, postoperative radiation therapy with a dose of 45 Gy was started and well tolerated. The patient was discharged from the hospital in stable condition.

## 3. Follow-Up Period

**Figure 7:**
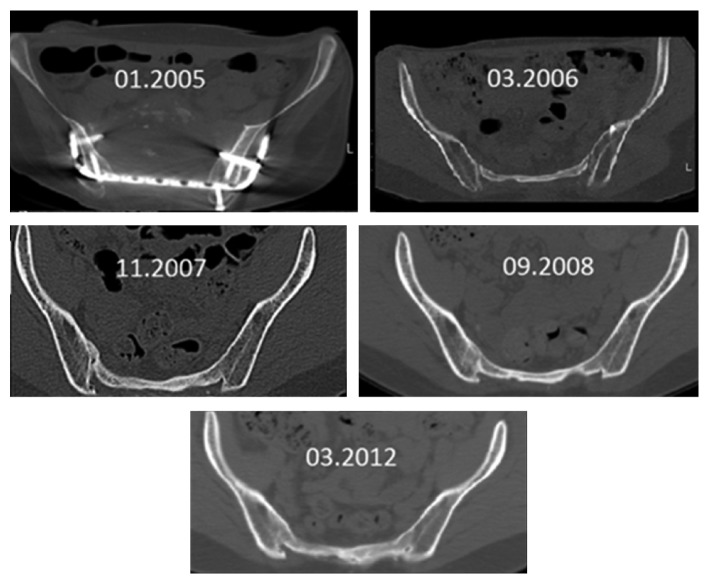
Sacral osteoneogenesis over the course of time in axial slices.

**Figure 8:**
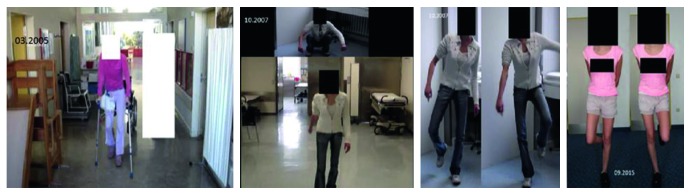
Improvement of gait and neurological status over time.

**Figure 9:**
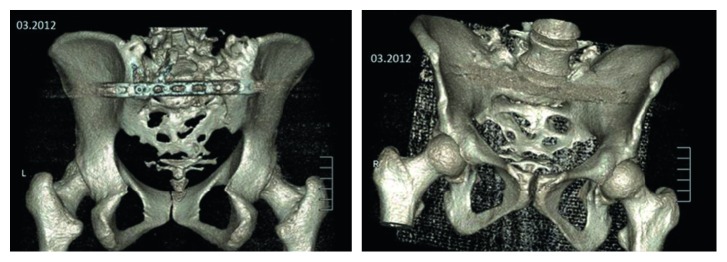
3D reconstruction of the “neosacrum.”

**Figure 10:**
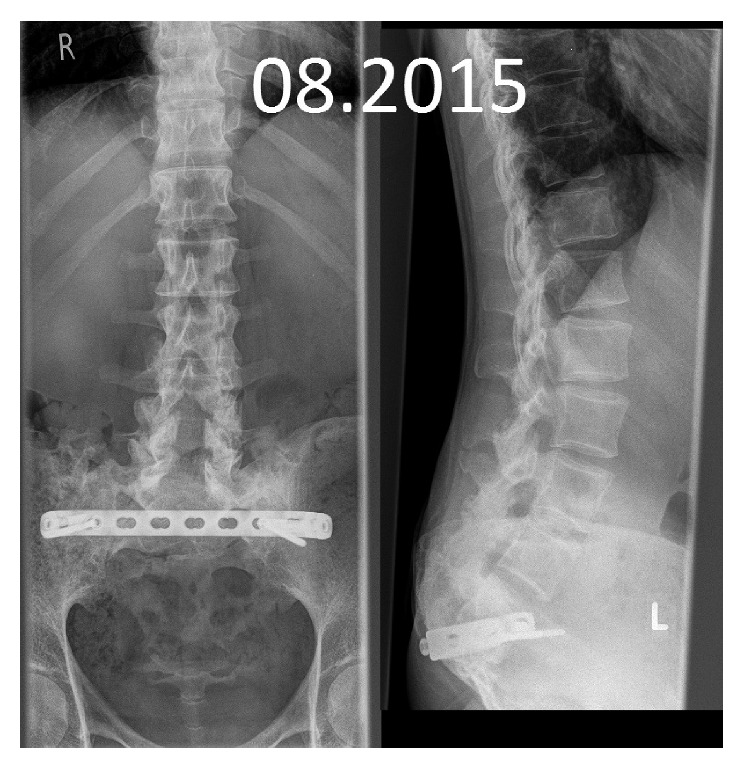
X-ray control in the lower spine.

The follow-up period was 12 years. At regular intervals, radiological follow-up examinations were carried out by means of CT, which documented the new formation of the sacrum, including the neuroforamina, initially as a thin bone margin in the area of the former sacrum up to the formation of a new sacrum (Figure 7). Neurologically, an incomplete paraplegia remained, with preserved motor function and sensitivity below L5/S1 with a clinically not relevant peroneal paresis on the right, discrete paresthesia on the right lateral thigh, and distal lower limbs as well as a neurogenic bladder dysfunction. Crutches were necessary only for longer distances. The gait was almost completely normal. The one-leg stand was possible on both legs as well as going into a squat position (Figure 8). Over the time, 3 operations including partial implant removal due to implant loosening including the USS as well as a removal of exostosis on both spina iliaca posterior superior were necessary. The radiological controls show an almost complete sacral osteoneogenesis with a stable fusion of the new sacrum with the posterior pelvic ring (Figures 9 and 10).

## 4. Discussion

Ewing sarcoma is a highly malignant bone tumor. Treatment options are radiation and chemotherapy as well as the surgery. The combination of these procedures increases the survival rate by 15–20% [1, 4]. The single use of radiation therapy leads depending on the location and size of the tumor to an increased local recurrence risk so that usually a combination of all 3 treatment options is favored. Recent studies by Hesla et al. suggested that local radiation therapy alone appears to result in good local tumor control and may be the treatment of choice for sacral tumors [2]. Nevertheless, the definitive treatment of an Ewing sarcoma is an individual decision. Since an en bloc resection was impossible, a dissection and therefore intralesional tumor resection were performed, so a follow-up radiotherapy was mandatory. The surgical access of the guide biopsy must be preoperatively selected on the basis of tumor localization and spread so that no contamination with tumor cells occurs during the subsequent definitive surgical treatment. In addition to R0 tumor resection, the aim is to maintain the best possible neurological status. An en bloc resection often leads to sacrifice of the sacral nerves and/or the vessels, in particular the iliac artery [5, 6]. A multisegmental intralesional tumor resection has an increased risk of metastasis and recurrence [7]. The extent of the iatrogenic-caused neurological damage cannot be assessed preoperatively [5, 8]. Frequent neurological disorders are muscle weakness or paralysis of the extremities and loss of control of intestines with incontinence and sexual dysfunction. The nerves S1–S3 supply the bladder, the intestine, and the rectum, so their preservation should always be achieved if possible. Cases were described in which there was either a complete or partial recovery of the bladder and colon function. A recovery of the sexual function is also possible [7, 9]. Biomechanically, the sacrectomy leads to considerable rotational and translational instabilities of the pelvic ring. A subsequent defect reconstruction can be done, but does not necessarily has to be done. The defect reconstruction can be performed by allogenic or autologous bone grafting, and hybrid forms are also possible. The later reimplantation of the resected sacrum after extracorporeal radiation is also possible, but this procedure is associated with an increased risk of infection [10–18]. The functional outcome appears to be independent of whether a reconstruction of the defect has occurred or not. Nevertheless, a dorsal spondylodesis is recommended in order to achieve a faster mobilization and rehabilitation [19, 20]. However, since the soft tissue is always endangered, additional mechanical stimuli with potential impairment of the local perfusion should be avoided. For this reason, we renounce wearing a corset [21]. In the end, the cause for the sacral osteoneogenesis formation of the “neosacrum” remains unclear. We believe that several factors can be responsible for this phenomenon. On the one hand, the sacrectomy has resulted in a large “fracture” hematoma with excessive release of mesenchymal progenitor cells from the bone marrow. Those progenitor cells accumulated along the LCP which served as a guiding structure for the sacral osteoneogenesis. On the other hand, the procedure was performed intralesionally with fragmentation of the tumor, which inevitably leaves tissue remnants in the marginal areas, from which the osteoneogenesis could start. Another explanation, although purely speculative, is that the LCPs are osteoinductive materials that promote osteoneogenesis. In the course of time, there was a gradual overbuilding of the plate with bones [22]. Similar phenomena are known after osteotomies or fractures on long bones. Bier assumed in his 1905 published paper about the potency of the fracture hematoma for the formation of new bone that the hematoma can also be useful for the healing of the fractures of the bones due to the fact that it always takes place under the influence of exceedingly great quantities of blood between and around the fracture. These therefore represent the natural conditions for the healing of a bone fracture [23, 24]. Bier rescinded these procedures due to unreliable treatment results, as the neocallus developed uncontrollably and it often resulted in complications such as the formation of a nonunion. He postulated that the reason for the delayed formation of the callus is the removal of the bruise and nursing the wound. “Where a large bruise is present, there is considerable bone and connective tissue formation.” Within a very short time, a “neosacrum” was formed which resembled in form and function the original form of the sacrum with the characteristic triangular shape distally including the neuroforamina. The patient successfully completed the secondary school and completed an apprenticeship in a tire trade. Routine work such as desk work and longer sitting but also the wearing of tires is possible. Currently, there is a wish for children. To the best of our knowledge, this is the first described case with a new formation of the sacrum after complete sacrectomy in a patient with an Ewing sarcoma.
